# Antifungal Plant Defensins as an Alternative Tool to Combat Candidiasis

**DOI:** 10.3390/plants13111499

**Published:** 2024-05-29

**Authors:** Ekaterina I. Finkina, Olga V. Shevchenko, Serafima I. Fateeva, Andrey A. Tagaev, Tatiana V. Ovchinnikova

**Affiliations:** M. M. Shemyakin & Yu. A. Ovchinnikov Institute of Bioorganic Chemistry, The Russian Academy of Sciences, Miklukho-Maklaya Str. 16/10, 117997 Moscow, Russiaovch@ibch.ru (T.V.O.)

**Keywords:** plant defensins, candidiasis, antifungal drugs, drug resistance, fungi, *Candida albicans*, *Candida auris*, anticandidal activity, prevention of fungus adhesion, antibiofilm activity, immunomodulatory effects

## Abstract

Currently, the spread of fungal infections is becoming an urgent problem. Fungi of the *Candida* genus are opportunistic microorganisms that cause superficial and life-threatening systemic candidiasis in immunocompromised patients. The list of antifungal drugs for the treatment of candidiasis is very limited, while the prevalence of resistant strains is growing rapidly. Therefore, the search for new antimycotics, including those exhibiting immunomodulatory properties, is of great importance. Plenty of natural compounds with antifungal activities may be extremely useful in solving this problem. This review evaluates the features of natural antimicrobial peptides, namely plant defensins as possible prototypes of new anticandidal agents. Plant defensins are important components of the innate immune system, which provides the first line of defense against pathogens. The introduction presents a brief summary regarding pathogenic *Candida* species, the pathogenesis of candidiasis, and the mechanisms of antimycotic resistance. Then, the structural features of plant defensins, their anticandidal activities, their mechanisms of action on yeast-like fungi, their ability to prevent adhesion and biofilm formation, and their combined action with conventional antimycotics are described. The possible mechanisms of fungal resistance to plant defensins, their cytotoxic activity, and their effectiveness in in vivo experiments are also discussed. In addition, for the first time for plant defensins, knowledge about their immunomodulatory effects is also presented.

## 1. Introduction

Recently, fungal diseases have become a serious clinical problem. In 2022, in order to attract public attention and strengthen the global response to the spread of fungal infections, WHO published a list of the most dangerous fungal pathogens [1]. The highest priority has been given to pathogens that are the cause of severe invasive fungal diseases and often characterized by drug resistance. Among the four fungi of the highest critical priority are two pathogens of the *Candida* genus, namely *C. auris* and *C. albicans*. 

Some representatives of the *Candida* genus, in particular *C. albicans* and *C. auris,* are part of healthy human microbiomes of the large intestine, vagina, skin, and oral cavity; however, a weakened immune system contributes to the development of opportunistic infections. Oropharyngeal, vulvovaginal, cutaneous, and mucosal candidiasis often occur. According to statistics, 75% of women around the world have had vaginal candidiasis at least once in their lives [2]. At the same time, systemic infections, such as candidemia and invasive candidiasis, are rather less common, but are characterized by a high mortality rate, ranging from 20% to 50%, despite ongoing antifungal therapy [1,3]. Immunocompromised patients with chronic lung disease, tuberculosis, diabetes, HIV, autoimmune diseases, cancer, or COVID-19, as well as premature babies, are at high risk [1,4,5]. *C. albicans* continues to be the most common cause of superficial, as well as invasive, candidiasis worldwide [1,4]. *C. auris* is still rare, but it causes invasive candidiasis with a high mortality rate and hospital outbreaks due to unprecedented stability and multiple resistance to conventional antifungal drugs [1,6,7,8].

The prevention of *Candida* infection is realized by the complex of the host defense system involving innate and adaptive immunity. Pathogen recognition by surface and internal immune receptors, the activation of different signal pathways, and the production of cytokines/chemokines and antimicrobial peptides (AMPs), as well as the interaction of various cells, including macrophages, dendritic cells, neutrophils, and T-lymphocytes, take place. However, a decrease in immune defense or/and an increase in fungus pathogenicity may lead to the development of superficial candidiasis or, in more serious cases, fungal invasion and systemic infection. As shown, the gastrointestinal tract is often the site of the penetration of *C. albicans* into the bloodstream [9]. Such virulence factors of *C. albicans* as the ability to adhere and switch morphology from yeast to the hyphal form, and vice versa, to form biofilms and penetrate into the bloodstream, play an important role in the pathogenesis of invasive candidiasis [3].

Currently, the prevention of the development of invasive candidiasis in patients with chronic superficial candidiasis is impossible, due to the lack of vaccines. At the same time, the list of drugs that can be used against superficial and invasive infections caused by *Candida* spp. is very limited. Antimycotics of three main groups with different mechanisms of antifungal action are used in clinical practice. Polyenes bind the component of the fungal cell membrane ergosterol, which leads to the disruption of the integrity of the plasma membrane [10]. Polyene amphotericin B (AmB) is one of the most powerful antifungal compounds, which, however, is characterized by a high toxicity, a low solubility, and poor oral bioavailability [11,12]. Azoles, including fluconazole and voriconazole, inhibit the enzymatic activity of lanosterol 14-α-demethylase, which is involved in the biosynthesis of ergosterol [13]. Echinocandins, in particular caspofungin and anidulafungin, inhibit the fungal-specific enzyme (1,3)-β-D-glucan synthase, which takes part in the biosynthesis of the cell wall component β-1,3-glucan [14]. The pyrimidine analog flucytosine inhibiting DNA and RNA synthesis in yeast-like fungi is also used as a part of combination therapy [10].

Unreasonable antimycotic administration and violations of the drug dosage regimen lead to the spread of antifungal drug resistance [15]. Azole-resistant strains of *Candida* spp., especially of *C. auris*, are now increasingly common, often due to point mutations in the *ERG11* gene encoding lanosterol-14α-demethylase [16] or the overexpression of this gene or other genes encoding transmembrane transporters CDR1, CDR2, and MDR1, which remove antifungal drugs from *Candida* cells [17]. The mechanism of the resistance development to AmB most often involves changes in the sterol composition of the fungal cell membrane, due to mutations in the *ERG11* gene and other *ERG* genes [18,19,20]. In some cases, cross resistance to azoles and polyenes occurs [18]. Resistance to echinocandins often results from point mutations in the *FKS1* and *FKS2* genes, which encode the enzyme (1,3)-β-D-glucan synthase [21]. Resistance to flucytosine develops quickly as a result of defects in the enzymes involved in its metabolism to 5-fluorouracil [10].

Thus, the search for new drugs for the treatment of superficial and systemic candidiasis, including those caused by resistant strains of fungi, is a very urgent task to date. Plant defensins can serve as prototypes of new antimycotics.

## 2. Plant Defensins

Defensins are a conserved class of cationic AMPs found in animals, plants, and fungi. Despite the low level of amino acid sequence homology, most defensins have a compact spatial structure, stabilized by three or four disulfide bonds, and are characterized by pronounced antimicrobial activity [22].

Defensins perform defense functions in plants, and the activation of their synthesis occurs under biotic and abiotic stresses. Most of them are secretory peptides synthesized as prepeptides with signal and mature domains, as shown, for example, for Lc-def from germinated seeds of lentil [23]. The precursors of some plant defensins, for instance, NaD1 from the flowers of ornamental tobacco, contain an additional C-terminal domain, which targets the defensin to the vacuole to protect the host cell against the antimicrobial peptide [24]. Plant defensins have a wide spectrum of biological activity, and, in a number of works, are considered as objects for the creation of new antiviral [25] and anticancer [26] drugs, as well as disease resistant plants [27]. However, in this review, they are described in the context of their potential use as new antifungal agents for the treatment of candidiasis.

### 2.1. Structural Features

**Figure 1:**
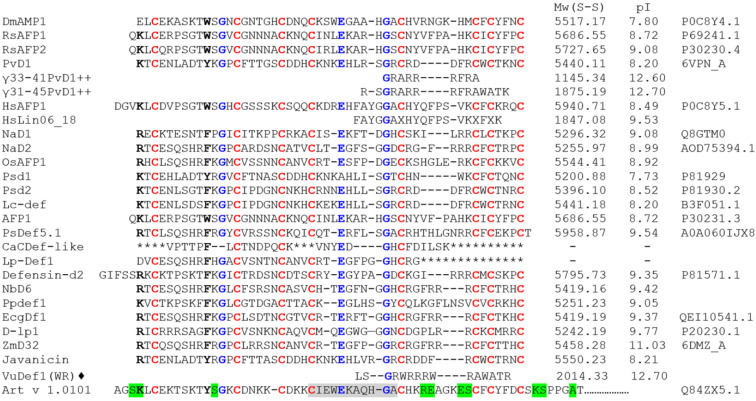
Amino acid sequences of plant defensins and their synthetic analogs possessing activity against fungi of the *Candida* genus. The amino acid sequences of the following peptides are given in: γ33-41PvD1++ and γ31-45PvD1++ [35]; HsLin06_18 [36]; OsAFP1 [37]; CaCDef-like [38]; Lp-Def1 [39]; NbD6 [40]; Ppdef1 [41]; Javanicin [42]; ♦ A_42_,_44_R_37_,_38_W_36,39_γ_32–46_*Vu*Def(WR) [43]. Art v 1 structure, the defensin-like allergen of mugwort pollen, is also present for comparation. Cysteine residues are red, conservative amino acids are blue, and homologous amino acids are bold. Amino acids included in the IgE-binding epitopes [44] and the dominant T-cell epitope [45] of Art v 1 are highlighted in green and gray, respectively. X—α-aminobutyric acid. *—unknown amino acid. The molecular weight (Mw) and isoelectric point (pI) of peptides are also presented.

**Figure 2:**
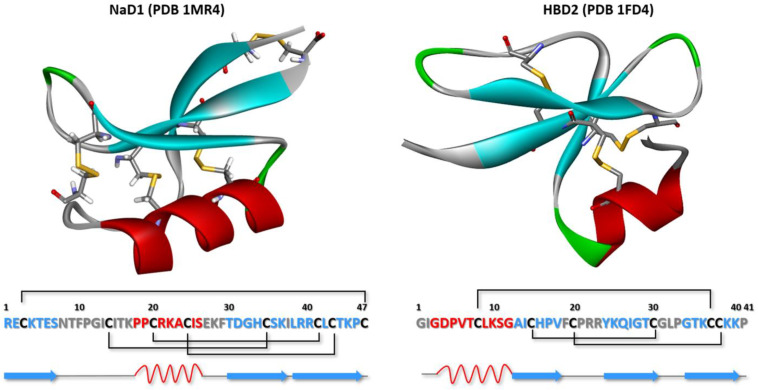
Structural organization of plant defensin NaD1 from flowers of ornamental tobacco (PDB 1MR4) [46] and human β-defensin HBD2 (PDB 1FD4) [47]. α-Helices and β-sheets are colored red and blue, respectively. The formation of disulfide bonds in the structures of defensins is also shown.

Plant defensins consist of 45–54 amino acid residues and, as a rule, have a basic isoelectric point [28]. Most plant defensins have a conserved tertiary structure consisting of one α-helix and three antiparallel β-strands that are linked by four disulfide bonds to form a cysteine-stabilized αβ (CSαβ) motif and βαββ fold [29,30]. Despite the uniformity of spatial organization, the structure of plant defensins lacks conserved regions (Figure 1) [30,31]. There is an opinion that plant, insect, and vertebrate β-defensins are closely related, due to structural similarities [29]. In particularly, human β-defensins, HBD1 and HBD2, have a spatial structure similar to that of plant defensins, also represented by α-helix with a triple-stranded anti-parallel β-sheet but forming αβββ fold (Figure 2) [29,32]. Plant defensins that contain an additional C-terminal glycosylated proline-rich domain were found in the pollen of some plants (Figure 1) [33,34].

Plant defensins, unlike many other classes of AMPs, demonstrate high stability, due to their compact structure stabilized by disulfide bonds. Plant defensins can withstand high temperatures and boiling [37,48], and retain their structure in strongly acidic solutions [48,49]. These peptides are resistant to proteolytic enzymes of the human gastrointestinal tract [49,50] and serum [37] and are not degraded by proteases of *C. albicans* [49].

### 2.2. Anticandidal Activity

As described above, plant defensins perform a protective function in plants consisting of the first line of plant antimicrobial defense. Unlike vertebrate defensins, which have a membranotropic effect and are characterized by a wide spectrum of antimicrobial activity, inhibiting the growth of Gram-positive and Gram-negative bacteria and fungi, plant defensins act mainly against fungi, which are the main cause of plant infection diseases. The latest data show that plant defensins are effective not only against phytopathogens, but also against human pathogenic filament and yeast-like fungi. The reason for the selectivity of antimicrobial action is the targets of plant defensins, which are specific lipid components of the fungal cell wall and membrane, but not in all cases. This will be discussed in more detail in the next sections.

#### 2.2.1. Activity against Planktonic Yeast-like Cells

Defensins from various plants have been shown to be effective against different species of the *Candida* genus (Table 1). Due to the fact that *C. albicans* and *C. auris* are high-priority pathogens, the activity of plant defensins against these species is of the greatest interest. To date, a lot of data have been provided on the activity of plant defensins and their shortened analogues against collection strains and clinical isolates of *C. albicans*. For example, DmAMP1 from *D. merckii*, tobacco NaD1, rice OsAFP1, radish RsAFP2, and maize ZmD32 inhibit fungal growth at micromolar concentrations. These peptides effectively kill yeast cells in viability tests and show fungicidal action. Other defensin like *Phaseolus vulgaris* PvD1, lentil Lc-def, barley D-lp1, CaCDef-like from *C. annuum*, pea Psd1, and Psd2 act fungistaticly in higher concentrations. It is worth noting that the variety of conditions for testing antifungal activity (culture media, the starting cell concentration, incubation time, and temperature) certainly affects the MIC values and complicates a comparative analysis (Table 1).

At the same time, data on the activity of plant defensins against another type of the highest priority fungus—*C. auris*—as well as against resistant and multi-resistant strains of fungi, are quite limited. Activity against *C. auris* has been shown for NaD1, ZmD32, and D-lp1 from *H. vulgare*. Javanicin from *S. javanica* was found to be equally effective against both sensitive and azole-resistant strains of *C. albicans* [42]. At the same time, D-lp1 affected the growth of amphotericin B-resistant *C. auris* at higher concentrations than that of the growth of the sensitive strain of the fungus [68]. The activity against other species of fungi of the *Candida* genus that cause candidiasis, including *C. glabrata*, *C. krusei*, *C. parapsilosis, C. tropicalis*, *C. buinensis*, *C. guilliermondii,* and *C. dubliniensis,* has also been shown for a number of plant defensins. It is interesting to note that synthetic peptides based on the γ-core motif from PvD1 more effectively inhibit the growth of *C. buinensis* than *C. albicans* (Table 1) [35].

#### 2.2.2. Mechanisms of Antifungal Action

As mentioned above, the structure of plant defensins lacks conserved regions, which apparently determines the diversity of their targets. Plant defensins have different complex mechanisms of antifungal action, but, most often, their targets are the components of the fungal cell membrane. The total positive charge of plant defensins provides initial electrostatic interaction with negatively charged components of the fungal cell wall and membrane [28].

The cells of yeast-like fungi and humans differ from each other in the composition of their membranes. In general, yeast membranes are more negative, due to the higher content of neutral and anionic phospholipids–phosphatidylinositol (PI) and phosphatidic acid (PA) instead of neutral phosphatidylcholine (PC), which is rich in human cell membranes. Yeast sphingolipids, particularly mannosyldiinositolphosphorylceramide (M(IP)2C), and glucosylceramides (GlcCers) are structurally different from those of mammals. Yeast cell membranes contain ergosterol instead of cholesterol, which is the main sterol of human cell membranes [69]. These differences largely determine the effectiveness of plant defensins and their low toxicity.

Some plant defensins interact with fungal-specific membrane lipids, in particular, M(IP)2Cs and GlcCers. However, the targets of other plant defensins are such universal lipid components as PA and phosphatidylinositol-4,5-diphosphate (PI(4,5)P2), located on the inner surface of the plasma membrane (Table 2). For instance, fungal GlcCers are the target for AFP1 from *B. juncea* [63], RsAFP2, Psd1, Psd2, and PvD1 (Table 2). It has been found that RsAFP2 does not interact with soybean or human glucosylceramides [51]. Moreover, *C. glabrata*, whose cell membrane does not contain GlcCers, unlike *C. albicans*, is resistant to RsAFP2-induced membrane permeabilization and cell death [24]. On the contrary, the absence of GlcCers in *C. albicans* strains deficient in the glucosylceramide synthase gene did not abolish the antifungal activity of Psd1 [61]. This occurs due to the fact that another target of Psd1 is ergosterol [70]. Psd1 has a much lesser effect on small unilamellar vesicles containing cholesterol than ergosterol, which is a possible explanation for the low toxicity of the peptide to mammalian cells [70]. The ability to bind different phosphatidylinositol phosphates (PIPs) and/or PA has been shown for HsAFP1 from *H. sanguinea*, NaD1, NaD2, ZmD32, OsAFP1, and Psd2 (Table 2). For instance, PI(4,5)P_2_ has been identified as a fungal target for NaD1, which forms an oligomeric complex with this lipid (namely oligomer of 7 dimers of NaD1 with 14 molecules of PI(4,5)P_2_) and causes membrane lysis [24,71]. It has been shown that OsAFP1 binds to PIPs, especially PI(3)P [72].

The question of how plant defensins penetrate the thick cell wall of fungi still remains open. One explanation is that some targets of plant defensins are also present in fungal cell walls. For example, it has been shown that PI(4,5)P_2_ binding is not essential for the antifungal activity of NaD1, whose mechanism of action is very complex [85]. It turns out that NaD1 interacts with such components of the cell wall as β-glucan and chitin [86].

It is believed that plant defensins may realize their anticandidal activity by triggering signaling cascades in fungal cells, which leads to ROS generation [87], mislocalization of septins, and the accumulation of ceramides [88]. For instance, NaD1 causes not only membrane permeabilization [89], but also oxidative damage [52]. However, the cytotoxic mechanism of NaD1 is membranolytic, rather than apoptotic [90]. The induction of plasma membrane permeabilization and the production of ROS is also characteristic of other plant defensins, such asPvD1, AFP1, So-D2 from spinach, CaDef2.1, and CaDef2.2 from *C. annuum* [57,63,65,91]. Moreover, PvD1 is able to cause the disorganization of both the cytoplasmic contents and the plasma membrane and inhibit glucose-stimulated acidification of the growth medium by yeast cells and filamentous fungi [57]. HsAFP1 induces the generation of ROS and causes DNA fragmentation, which are key markers of cell apoptosis [92]. It has also been found that the genes involved in pathways associated with GPI-anchored proteins, cation homeostasis, autophagy, and the cell cycle, are up- or down-regulated in *C. albicans* upon HsAFP1 treatment [93]. On the other hand, the presence of intracellular target cyclin F, which is an important component of the cell cycle, has been demonstrated for Psd1 using *Neurospora crassa* [61]. A detailed analysis of the key structural elements and key amino acids responsible for the antifungal activity of plant defensins is given in a recent review [94].

#### 2.2.3. Effect of Salts on Antifungal Activity

It is known that many AMPs lose their activity in the presence of salts at physiological concentrations [95,96]. This may be due to the weakening of electrostatic interactions between cationic AMPs and the anionic surfaces of microbial cells, the cell wall, and the cell membrane in the presence of monovalent and divalent cations. In addition, it has been demonstrated that a shift to a high salt concentration induces a tightening of the cell wall matrix of *C. albicans* [40]. As shown, the anticandidal activity of many, but not all, plant defensins and their synthetic analogs decreases in the presence of different salts (Table 2). For example, sodium chloride in a physiological concentration inhibits the activity of NaD1, preventing the interaction and penetration of this defensin through the yeast cell wall [86]. At the same time, DmAMP1, which, unlike NaD1, binds to M(IP)2C in the cell wall and/or membrane and does not interact with β-glucan and chitin, is salt-tolerant. Due to the ability of NaD1 to bind liposomes at salt concentrations at or above 100 mM, it is assumed that the increase in ionic strength interferes with the ionic interaction between this defensin and components of cell wall, such as β-glucan [86]. On the contrary, lentil Lc-def is able to bind anionic vesicles only under low-salt conditions, but not at the physiological ionic strength (100 mM NaCl) [97]. Other salt-resistant antimicrobial plant defensins are ZmD32 and NbD6, which do not lose their activity in the presence of NaCl, MgCl_2_, or CaCl_2_ [40]. The authors suggest that this is due to the high positive charge of these peptides (Table 1) [40].

#### 2.2.4. Prevention of Yeast-like Cell Adhesion and Antibiofilm Activity

As noted above, the ability to adhere to and form biofilms are important virulence factors for fungi of the *Candida* genus. The attachment of yeast to host surfaces, such as buccal and intestinal epithelium, is a critical parameter for the colonization and development of candidiasis. The anti-adherence properties of D-lp1 against *C. auris* have been demonstrated using buccal epithelial cells. This defensin, in concentrations of 0.19 mg/mL (0.25 × MIC), 0.39 mg/mL (0.5 × MIC), and 0.78 mg/mL (MIC), inhibited the attachment of yeast-like cells to the epithelial cells at 4.72%, 38.62%, and 73.52%, respectively [68].

As known, biofilms are characterized by high heterogeneity and are covered with a matrix that prevents the penetration of antifungal drugs, significantly reducing their effectiveness. Some plant defensins, for example, RsAFP2, HsAFP1, HsLin06_18, Psd1, and D-lp1, are able to prevent biofilm formation, but in higher concentrations than those of their MICs (Table 2). In contrast, So-D2 effectively prevents the formation of *C. albicans* biofilm in a low concentration [65]. As described, RsAFP2 prevents *C. albicans* biofilm formation, due to its ability to block the yeast-to-hypha transition [77,88]. The ability to eradicate the mature biofilms of fungi of the *Candida* genus has also been shown for some, but not all, plant defensins investigated, such as ZmD32 and D-lp1 [40,68,84]. However, the difference in the activity of plant defensins may also be due to the differences in the conditions under which the fungal biofilms were obtained (Table 2).

#### 2.2.5. Synergistic Effect

The use of combination therapy, in which two or more antifungals enhance each other’s activity, is one of the strategies used for optimizing the treatment of candidiasis, which has many advantages over monotherapy. The synergistic effect of the combinations of plant defensins with conventional antimycotics of the echinocandin and polyene groups, as well as inhibitors of proteolytic enzymes, has been shown (Table 2). The synergistic effect against *C. albicans* with caspofungin has been shown for such defensins as NaD1, DmAMP1, RsAFP1, RsAFP2, HsAFP1, and its linear 19-mer analog HsLin06_18. Decreasing the 1,3-β-glucan levels with echinocandins apparently facilitates the penetration of plant defensins through the yeast cell wall to the cell membrane, where the targets of their action are located [86]. Moreover, it has been shown that caspofungin can increase the PI(4,5)P2 levels in the fungal membrane, thereby facilitating PIP-dependent internalization of plant defensins [36]. Synergistic effects have also been observed between some plant defensins (RsAFP1, RsAFP2, and HsAFP1) and AmB, which binds ergosterol and forms pores in the membrane and also causes oxidative stress in the yeast cell. Interestingly, synergistic effects of combinations of plant defensins with caspofungin or AmB have been observed in some cases only against planktonic cells. In other cases, combinations act effectively against the formation and eradication of biofilms, but not against planktonic cells (Table 2). Bovine pancreatic trypsin inhibitor has been shown to act in synergy with NaD1 and DmAMP1, but the synergistic activity is not related to its protease inhibitory activity [59].

#### 2.2.6. Fungal Resistance to Plant Defensins

The development of antimicrobial resistance to AMPs is less common compared to that of antimicrobial drugs, since the targets of AMPs are diverse and are often components of the cell membrane, the change or alteration of which can lead to the disruption of normal cellular function. However, in vitro experiments have shown that resistance to AMPs can still develop [98]. The possible mechanisms for the development of resistance in yeasts and yeast-like fungi have been shown for several plant defensins.

Using screening of the *Saccharomyces cerevisiae* deletion mutant library, yeast strains resistant to HsAFP1 have been identified. It has been revealed that mutations in the genes taking part in mitochondrial functionality, vacuolar acidification, protein sorting and vesicular transport, gene expression, and DNA repair provide yeast resistance to HsAFP1 [92]. Serial passages with *S. cerevisiae* were performed to examine the evolution of resistance to NaD1. The development of tolerance to NaD1 takes place, but it occurs more slowly compared to that of caspofungin. Genome sequencing has revealed mutations in 12 genes that are involved in resistance and participate in such processes as cell wall formation, membrane transport, and signaling functions [99]. Multidrug-resistant *C. albicans* ATCC 64124 has been used for the investigation of the development of yeast resistance to So-D2. No fold changes in the MIC of the peptide have been observed, indicating the low resistance potential of this plant defensin [65].

### 2.3. Immunomodulatory Effects

As described above, candidiasis poses a threat primarily to immunocompromised patients. For example, the decrease in IL-17 levels taking place in HIV-infected patients and patients with genetic defects of IL-17 immunity predisposes them to the development of mucocutaneous candidiasis [100,101]. On the other hand, uncontrolled inflammation can lead to tissue damage and the progression of the infection [102]. It is known that the effectiveness of candidiasis treatment increases with the use of combination therapy, including, in addition to antifungals, immunomodulatory drugs [103]. Therefore, the search for new antimycotics with immunomodulatory properties is of great interest.

It is well known that human AMPs, particularly cathelicidin LL-37, defensins, and histatin, play a complex role in host defense, exhibiting not only antimicrobial activity, but also immunomodulatory effects in much lower concentrations. In particular, human defensins help to prevent the development of infection acting on various immune cells directly or in complex with proteins, nucleic acids, and carbohydrates, taking part in chemotaxis and phagocytosis, inducing or suppressing inflammatory responses. As shown, human defensins implement their immunomodulatory action through a wide range of immune receptors, including different Toll-like receptors (TLRs) and chemokine receptors [104]. It is worth noting that two AMPs that are in clinical trials as new agents for candidiasis treatment, namely histatin-derived peptide PAC113 and melanocyte-stimulating hormone-derived peptide CZEN-002, exhibit, in addition to anticandidal activity, immunomodulatory properties [105].

The recent data show that plant defensins possess not only antifungal activity, but also the ability to modulate the immune response. To date, this information is quite limited (Table 3). Nevertheless, immunomodulatory effects have been shown for γ-thionin from *C. chinense*, Psd1, Lc-def, and two synthetic peptides—EgK5 (designed analogue of plant defensins from grapevine and oil palm) and SolyC (γ-motif of tomato defensins).

It has been shown that plant defensins have immunomodulatory, mainly pro-inflammatory, effects on epithelial cells, which are the first line of defense against pathogens (Table 3). Psd1 upregulates the expression of gene coding human β-defensin-2 (HBD2), IL-1β, and IL-8/CXCL8, as well as increases the production of IL-6 and IP-10 in intestinal-like epithelial cells Caco-2 [79]. γ-Thionin upregulates the expression of gene coding membrane receptor TLR2 and cytokines (TNFα, IL-1β, and IL-10), activates the transcriptional factors of inflammatory response (E2F-1, EGR, CBF, AP-1, and others), and increases NO production in bovine mammary epithelial cells (bMECs) [107,108]. The immunomodulatory effects of plant defensins on epithelial cells also persist under infection. Thus, the immunomodulatory effects of Psd1 on Caco-2 cells and γ-thionin on bMECs infected by *C. albicans* or *S. aureus*, respectively, are generally the same as those without infection [79,107,108]. It is worth mentioning that γ-thionin significantly inhibits *S. aureus* internalization into bMECs. Since this peptide does not exhibit antibacterial activity, this effect is probably associated with its immunomodulatory action [107].

It has also been shown that plant defensins are able to pass through the different cell types. Ppdef1 from *P. pentandra* rapidly and efficiently penetrates human nail plates consisting of keratinized epidermal cells, making it an excellent candidate for a novel topical treatment of onychomycosis [66]. At the same time, Psd1 is able to pass through a Caco-2-cell monolayer simulating intestinal epithelium, which makes its further interaction with immune and endothelial cells possible [79].

It has been shown that Psd1, as well as Lc-def, affects the production of a broad range of both pro- and anti-inflammatory cytokines/chemokines and growth factors by different types of immune cells (Table 3). It is interesting to note that Psd1, as well as Lc-def, increases almost the same immune factors in both cultures, monocyte-derived dendritic cells (moDCs), and monocytes [49,79]. Moreover, Lc-def induces the production of IL-17 by regulatory CD4^+^ FoxP3^+^ T cells (Tregs), which play a critical role in anti-candida immunity [49]. Peptide solyC affects THP-1 stimulated with lipopolysaccharide but has no effect on unstimulated cells [110]. In addition, γ-thionin and PaDef from *P. americana* have an effect on endothelial cells [109].

The influence of Psd1 on epithelial–immune cell crosstalk using Caco-2/immune cells co-culture upon the *C. albicans* infection has also been investigated (Table 3). Virtually no effects on the immune response in Caco-2/immune cell co-cultures with Psd1 were shown, in contrast to the direct stimulation of immune cells by the pea defensin. *C. albicans* induces a pronounced host defense response in Caco-2/immune cell co-cultures; however, the cytokine responses are different in the presence of dendritic cells or monocytes. At the same time, Psd1 remodulates the immune response in both of these co-cultures upon infection, leveling the stimulating or, conversely, inhibitory effects of the pathogenic fungus [79].

The immunomodulatory effects of plant defensins has also been demonstrated in experiments in vivo. Psd1 does not only inhibit the formation of lung metastasis nodules in a murine model, but also decrease the number of inflammatory cells in lung tissues, possibly due to its immunomodulatory action [80]. Synthetic peptide EgK5 was effective in treating such autoimmune diseases as rheumatoid arthritis and atopic dermatitis in rat models [106].

**Figure 3:**
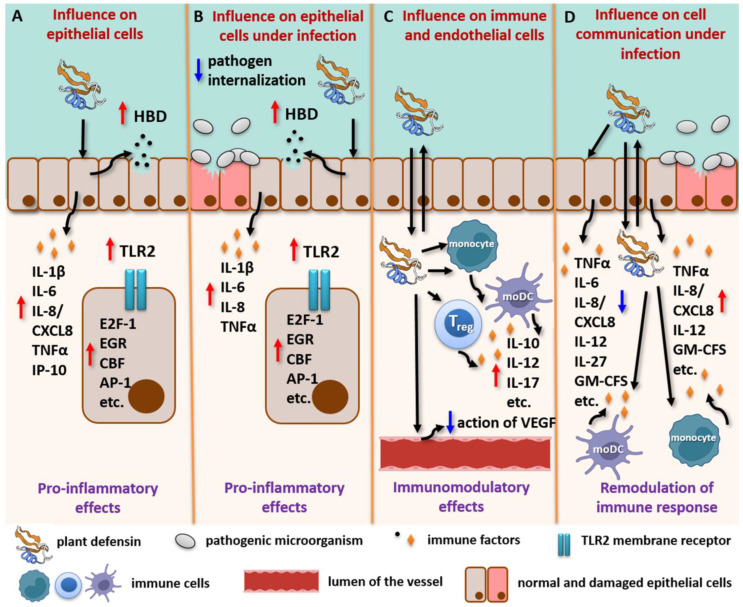
Possible immunomodulatory effects of plant defensins in low concentrations without and upon infection. (**A**) Plant defensins have a mainly pro-inflammatory effect on the epithelium, increasing the production of AMPs and pro-inflammatory cytokines/chemokines, as well as the abundance of PRRs and activity of the transcription factors of inflammatory response. (**B**) Under infection, plant defensins have a similar pro-inflammatory effect on the epithelium and also reduce the internalization of the pathogen. (**C**) Plant defensins are able to penetrate the epithelial barrier and affect various types of immune cells, increasing the production of pro- and anti-inflammatory cytokines/chemokines and growth factors. Plant defensins also affect endothelial cells interfering with the VEGF pathway. (**D**) Under infection, plant defensins affect epithelial–immune cell crosstalk, leveling the stimulating or, conversely, inhibitory effects of the pathogenic fungus and thereby remodulating the immune response. moDC—monocyte-derived dendritic cell, Treg—regulatory CD4^+^ FoxP3^+^ T cell; E2F-1, EGR, CBF, AP-1, etc.,—transcriptional factors of inflammatory response; TLR2—cell-membrane-associated receptor; HBD—human β-defensin; VEGF—vascular endothelial growth factor; IL-1β, IL-6, IL-8/CXCL8, IL-10, IL-12, IL-17, IL-27, IP-10, TNFα, and GM-CSF—pro- and anti-inflammatory cytokines, chemokines, and growth factors. Blue and red arrows indicate the upregulation/downregulation of genes encoding the corresponding proteins or an increase/decrease in the abundance of the appropriate proteins or an inhibitory/activating effect of plant defensins on some processes.

Summing up all of the data presented, we can conclude that plant defensins certainly have an immunomodulatory effect (Figure 3). They act on epithelial cells, are able to penetrate the epithelial barrier, and affect various types of immune and endothelial cells. Plant defensins influence the expression of the genes of pattern-recognition receptors (PRRs) and AMPs; impact the production of pro- and anti-inflammatory cytokines, chemokines, and growth factors; and also activate the transcription factors of inflammatory response. Moreover, under infection, plant defensins are able to remodulate the immune response induced by pathogens. These data demonstrate the need for further studies of the possible influence of plant defensins on various immune processes. The questions about how plant defensins exert their immunomodulatory effect and whether it will contribute to the prevention and treatment of candidiasis remain open.

### 2.4. Cytotoxic and Allergenic Properties

Unlike animal AMPs, plant defensins are generally characterized by low toxicity, but the situation is different for various representatives of this class (Table 2). Erythrocytes, epithelial, immune, and other types of mammalian cells have bene used to study the cytotoxicity of plant defensins. Such plant defensins as Psd1, Psd2, RsAFP1, RsAFP2, HsAFP1, and PvD1 do not cause hemolysis of erythrocytes and do not exhibit cytotoxic properties at concentrations significantly higher than their MICs. On the other hand, NaD1, ZmD32, D-lp1, and So-D2 exhibit hemolytic activity and cytotoxic properties in high concentrations.

As shown, some defensins are pollen and food allergens [28,32]. Thus, allergenic defensins, the structure of which contains an additional C-terminal glycosylated proline-rich domain, have been isolated from the pollen of mugwort and other plants. The most clinically significant defensin-like allergen is Art v 1 from mugwort pollen (Figure 1) [33]. Known conformational IgE-binding epitopes of Art v 1 are located in both defensin-like and proline-rich domains [111]. It is known that conserved structural regions, which correspond to B-cell epitopes, are present in the structure of cross-react allergens, in particular, plant lipid transfer proteins (LTPs) and homologues of the major birch pollen allergen Bet v 1 4 [112]. Nonetheless, there are no conservative regions in the structure of plant defensins, which makes the development of cross-allergic reactions unlikely. However, the question of whether other plant defensins, as well as the AMPs of different classes, can exhibit allergenic properties remains open.

### 2.5. In Vivo Experiments and Clinical Trials

Although the anticandidal activity of plant defensins in vitro has been widely demonstrated, their effectiveness in vivo has been shown in a very limited number of studies. It has been shown that RsAFP2 prevented the development of murine candidiasis. In this study, mice were inoculated intravenously with *C. albicans*, and defensin was administered 1 h later and every following 24 h after infection over 4 days. RsAFP2 was not inactivated by serum, was non-toxic to the mammalian cells, and significantly reduced the fungal burden in the kidneys of the infected mice [54].

*Galleria mellonella* was used as an infection model in another study. It was shown that PvD1 prolonged the survival of the larvae infected with yeast in lethal or sub-lethal concentrations, but this effect was dependent on the *Candida* species. PvD1 was most successful in the case of *C. buinensis* and *C. tropicalis* (survival rate > 80%) than for *C. albicans* and *C. parapsilosis* (survival rate < 20%) and also did not have a toxic effect on the larvae [56].

The antibiofilm action of HsLin06_18 and its combination with caspofungin was also investigated in vivo using a subcutaneous rat catheter model. *C. albicans* biofilms were formed inside catheter pieces in immunosuppressed rats. HsLin06_18, caspofungin, or a combination of the two were administered intravenously or subcutaneously immediately after infection and over 7 days after infection. A significant decrease of *C. albicans* biofilm formation on the catheters in the rats was observed only for the combination treatment. HsLin06_18 was not toxic for human cells but was probably not very stable under in vivo conditions [36].

Ppdef1, also known as HXP124 and pezadeftide, investigated by Hexima Ltd., is active in vitro against a number of clinically important fungal pathogens, including *Candida* spp. [41]. Pezadeftide, used topically in Phase 1 and 2 clinical trials (ACTRN12618000131257 and ACTRN12620000697987), is positioned as a topical treatment for onychomycosis causing by dermatophytes such as *Trichophyton rubrum* (https://hexima.com.au/ accessed on 26 May 2024) [66].

## 3. Conclusions

Defensins are AMPs ubiquitously present in various plant organs and tissues. These natural compounds play an important role in plant defense against infections, which are mainly caused by phytopathogenic fungi. Moreover, plant defensins also effectively act against human pathogens, including fungi of the *Candida* genus. This review presents a comprehensive analysis of the anticandidal potential of plant defensins.

Taking into account all of the summarized data, the following conclusions can be drawn: (1) Plant defensins have a compact structure stabilized by disulfide bonds, due to which they demonstrate a high resistance to heat, pH change, and proteolysis, unlike many other AMPs. (2) Plant defensins possess anticandidal activity against sensitive and resistant strains of yeast-like fungi and can act in synergy with conventional antimycotics. (3) These peptides are also able to counteract virulent factors of *Candida* spp. such as adhesion and biofilm formation. (4) The activity of some, but not all, plant defensins is reduced in the presence of salt at its physiological concentration. (5) Despite the uniformity of spatial organization, the primary structures of plant defensins lack conserved regions, which apparently determines the diversity of their targets and the mechanisms of antifungal action and reduces the risk of the emergence of resistant yeast-like fungi. (6) A low cytotoxicity is characteristic of many, but not all, representatives of this AMP class. (7) Recent evidence shows that plant defensins have an immunomodulatory effect, influencing various immune processes. These peptides affect the expression of the genes of PRRs and AMPs, the production of NO, pro- and anti-inflammatory cytokines, chemokines, and growth factors, the activity of inflammatory transcription factors, and also remodulate the immune response upon infection.

In summary, all of these taken together make plant defensins promising prototypes of new therapeutic agents for the prevention and treatment of candidiasis.

## Figures and Tables

**Table 1:** Anticandidal activity of plant defensins and their synthetic analogs.

Defensin	Source	*C. albicans*			*C. auris*	Other *Candida* Species	References
		MIC_50_	MIC_100_	MFC			
DmAMP1	*Dahlia merckii*, seeds	0.8 ^a^ µM	5 ^b^ µM	1.2% survival at a concentration of 10 µM	-	*C. glabrata*	[51,52,53]
RsAFP2	*Raphanus sativus*, seeds	-	2.5 ^b^ µM	10 µM	-	*C. krusei* *C. dubliniensis* *C. tropicalis* *C. parapsilosis*	[51,53,54]
PvD1	*Phaseolus vulgaris*, seeds	25–50 ^c^ µg/mL	MIC_89_ 18.4 ^d^ µM	-	-	*C. tropicalis* *C. buinensis* *C. parapsilosis* *C. guilliermondii*	[55,56,57]
γ_33–41_ PvD1^++^	Synthetic peptides based on the γ-core motif from PvD1	146.8–293.6 ^e^ µM	>293.6 ^e^ µM	-	-	*C. buinensis* MIC_100_ 36.7 ^e^ µM, MFC 73.4 µM	[35]
γ_31–45_ PvD1^++^		0–18.35 ^e^ µM	73.4 ^e^ µM	-	-	*C. buinensis* MIC_100_ 18.35 ^e^ µM	[35]
HsAFP1	*Heuchera sanguinea*, seeds	18 ^f^ µM	-	10 µM	-	*C. krusei*	[53,58]
NaD1	*Nicotiana alata*, flowers	1.6 ^g^ µM >10 ^h^ µM 3.6 ^i^ µM	2.5 ^g^ µM >10 ^a^ µM	MFC_50_ 15 µM	MIC_50_ 2.9 ^g^ or >10 ^h^ µM	*C. glabrata* *C. krusei* *C. parapsilosis* *C. tropicalis*	[40,52,59]
NaD2	*Nicotiana alata*, flowers	2.5–5 ^g^ µM >10 ^h^ µM	5 ^g^ µM >10 ^h^ µM	-	-	-	[40]
OsAFP1	*Oryza sativa japonica* (genome)	2 ^j^ µM	4 ^j^ µM	16 µM	-	-	[37]
Psd1	*Pisum sativum*, seeds	5–10 ^k^ µM	20 ^k^ µM	MFC_70,6_ 20 µM, but only MFC_48,7_ at a concentration of 200 µM	-	-	[60,61]
Psd2	*Pisum sativum*, seeds	6.9 ^l^ µM	MIC_90_ 20 ^l^ µM	-	-	-	[62]
Lc-def	*Lens culinaris*, germinated seeds	25–50 ^m^ µM	MIC_90_ 50 ^m^ µM	Fungistatic	-	*C. glabrata* *C. krusei*	[49]
AFP1	*Brassica juncea*, seeds	3–5 ^n^ µg/mL	10 ^n^ µg/mL	Cell viability decreased as the peptide concentration increased	-	-	[63]
PsDef5.1	*Pinus sylvestris*, seeds	6 ^o^ µM	15 ^o^ µM	-	-	-	[64]
CaCDef-like	*Capsicum annuum,* leaves	MIC_44_ 50 ^p^ µg/mL (there was no significant difference between 50, 100, and 200 µg/mL)	-	Fungistatic	-	-	[38]
Lp-Def_1_	*Lecythis pisonis,* seeds	MIC_38,5_ 10 ^q^ µg/mL	-	MFC_69,3_ 10 µg/mL	-	-	[39]
Defensin-d2 (So-D2)	*Spinacia oleracea,* leaves	-	7.5 ^r^ µg/mL	63 µg/mL	*-*	-	[65]
NbD6	*Nicotiana benthamiana*	1–2.5 ^g,h^ µM	2.5 ^g^ µM 5 ^h^ µM	-	-	-	[40]
Ppdef1	*Picramnia pentandra* (genome)	3.4 ^w^ µg/mL	30 ^x^ µg/mL	30 µg/mL	MIC_50_ 1.3 ^w^ µg/mL	*C. glabrata* *C. krusei* *C. tropicalis*	[41,66]
EcgDf1	*Erythrina crista-galli,* seedlings	0.1–0.2 ^s^ µM	1.5 ^s^ µM	1.5 µM	-	-	[67]
Javanicin	*Sesbania javanica,* seeds	50 ^t^ µg/mL	MIC_90_ 100 ^t^ µg/mL	100 µg/mL	-	-	[42]
		Fluconazole-resistant *C. albicans*					
		50 ^t^ µg/mL	MIC_90_ 100 ^t^ µg/mL	100 µg/mL			
ZmD32	*Zea mays*, endosperm	1.1 ^g^ µM 3.0 ^h^ µM	*-*	10 µM	MIC_50_ 3.4 ^g^ or 1.6 ^h^ µM	*C. glabrata* *C. krusei* *C. parapsilosis* *C. tropicalis*	[40]
A_42_,_44_R_37_,_38_ W_36,39_ γ_32–46_*Vu*Def (WR)	Synthetic peptides based on the region corresponding to the γ-core VuDef1 from *Vigna unguiculata* seeds	-	18.5 ^u^ µM	27.5 µM	-	*C. buinensis* *C. tropicalis*	[43]
D-lp1	*Hordeum vulgare,* seeds	-	47 ^v^ µg/mL	188 µg/mL	MIC_100_ 47 ^v^ µg/mL MFC 95 ^v^ µg/mL	*C. parapsilosis*	[68]
					Amphotericin B-resistant *C. auris*		
					MIC_100_ 78 ^v^ µg/mL MFC 156 ^v^ µg/mL		

MIC_50_ and MIC_100_—minimal inhibiting peptide concentrations required for 50% or 100% fungal growth inhibition, respectively; MFC—minimal fungicidal peptide concentration required for 100% fungal death. Growth conditions used: ^a^ ½ potato dextrose broth (PDB), 5 × 10^3^ cells/mL, incubation for 24 h at 30 °C; ^b^ PDB or PDB/yeast extract peptone dextrose (YPD); ^c^ Sabouraud broth (SB), 10^4^ cells/mL, 36 h at 28 °C; ^d^ PDB, 10^4^ cells/mL, 48 h at 30 °C; ^e^ SB 10^4^ cells/mL, 24 h at 30 °C; ^f^ RPMI-1640 medium (salt concentration 132.1 mM) with 0.5% DMSO, 10^6^ cells/mL, 24 h; ^g^ ½ PDB, cell culture with OD_600_ 0.0002, 24 h; ^h^ ½ PDB + 100 mM NaCl, cell culture with OD_600_ 0.0002, 24 h; ^i^ PDB, 5 × 10^3^ cells/mL, 24 h at 30 °C; ^j^ ½ PDB with 25 μg/mL of uridine, 10^5^ cells/mL, 14 h at 30 °C; ^k^ modified YPD, 10^4^ cells/mL, incubation at 25 °C until 50% of maximum fungal growth (growth curve OD_540_); ^l^ YPD, 10^4^ cells/mL, incubation at 25 °C until reaching OD_540_ 1; ^m^ ½ SB 10^4^ cells/mL, 24 h at 30° C; ^n^ ½ PDB, cell culture with OD_600_ 0.01, 16 h at 30 °C; ^o^ ½ LB, 10^4^ cells/mL, 18 h at 30 °C; ^p^ Sabouraud dextrose broth, 10^4^ cells/mL, 48 h at 30 °C; ^q^ SB, 10^4^ cells/mL, 36 h at 30 °C; ^r^ Mueller–Hinton broth (MHB), 10^3^ cells/mL, 48 h at 37 °C; ^s^ PDB,10^4^ cells/mL; ^t^ tryptic soy broth (TSB), 5 × 10^3^ cells/mL, 37 °C; ^u^ SB, 2 × 10^3^ cells/mL, 24 h at 30 °C; ^v^ RPMI-1640, 24 h at 35 °C; ^w^ YPD, 5 × 10^3^ cells/mL, 24 h at 30 °C; ^x^ ½PDB, 5 × 10^3^ cells/mL, 20 h at 30 °C. It is also worth considering the different species of *C. albicans* and *C. auris* used.

**Table 2:** Features of the most studied plant defensins with anticandidal activity.

Defensin	Possible Membrane Targets of Antifungal Action	Synergistic Activity against *C. albicans*	Activity against *C. albicans* Biofilms		Hemolytic and Cytotoxic Activity	References
			Inhibition of Biofilm Formation	Eradication of Mature Biofilms		
DmAMP1	Ergosterol M(IP)2C	Bovine pancreatic trypsin inhibitor (against planktonic cells)	-	-	-	[59,73,74,75]
RsAFP1	-	With caspofungin against biofilm formation and biofilm eradication, but not against planktonic cells	Not observed ^a^ up to 2 mg/mL	Not observed ^b^ up to 2 mg/mL	No hemolysis up to 500 µg/mL; not toxic to human HUVEC and human-skin-muscle fibroblasts up to 500 µg/mL	[76,77]
RsAFP2	GlcCer	With caspofungin against biofilm formation and biofilm eradication, but not against planktonic cells, with AmB against biofilm formation	+ ^a^ BIC_50_ 1.65 mg/mL	Not observed ^b^ up to 2 mg/mL		
HsAFP1	PA PI(3,4,5)P_3_ PI(3,4)P_2_ PI(3,5)P_2_ PI(4,5)P_2_ PI(3)P PI(4)P PI(5)P	With caspofungin against planktonic cells, biofilm formation and biofilm eradication, with AmB against biofilm formation	+ ^c^ BIC_50_ 11 μM	Not observed ^c^ up to 109 μM	Not toxic to HepG2 up to 40 μM	[58,78]
HsLin06_ 18 (linear 19-mer analog of HsAFP1)	PI(4,5)P_2_	With caspofungin against biofilm formation in the case of sensitive and caspofungin-resistant strains, increase activity in combination with anidulafungin	+ ^c^ BIC_50_ >2 μM	-	Not toxic to HepG2 cells up to 50 μM	[36]
NaD1	PI(3,4,5)P3 PA PI(3)P PI(4)P PI(5)P PI(3,4)P2 PI(3,5)P2 PI(4,5)P2 PS sulfatide cardiolipin	Caspofungin, bovine pancreatic trypsin inhibitor (against planktonic cells)	-	Not observed ^d^ up to 50 μM	At 50 µM, the percentage of hemolysis was 1.5%; at 12.5 µM, the percentage of AHDF cell viability was about 40%	[40,59,71]
ZmD32	PI(4)P PI(4,5)P PA PI(3,4,5)P3 PS cardiolipin	-	-	+ ^d^ BEC_50_ 10–15 μM	At 50 µM, the percentage of hemolysis was 1.9% and the percentage of AHDF cell viability 70%	[40]
Psd1	Ergosterol GlcCer	-	+ ^e^ No cell growth was observed at 10 × MIC (200 μM)	+ ^e^ Partial eradication of biofilm	Not toxic at a concentration of up to 50 μM to PBMCs, Beas-2B, HEK-293, R8, HSP, and CHO cells	[60,61,70,79,80]
Psd2	PS GlcCer cardiolipin PI(3)P PI(5)P ergosterol sulfatide POPC	-	-	-	No hemolysis up to 50 μM	[62]
PvD1	GlcCer	-	-	-	No hemolysis up to 30 μM, not toxic to HUVEC up to 100 μM; at 100 μM, the percentage of MCF 10A cell viability was 35–50%	[56,81,82,83]
Defensin-d2 (So-D2)	-	Antagonism with defensin-like bacteriocin actifensin from *Actinomyces* *ruminicola*	+ ^f^ BIC_50_ 7.5–15 µg/mL	-	At 128 × MIC (985 µg/mL), the percentage of hemolysis was 2.89%	[65]
D-lp1	-	-	*C. auris* biofilms		At MIC and MFC, the percentage of hemolysis was 9.55% and 14.72%, respectively. At MIC, cytotoxic activity to Caco-2 was absent	[68,84]
			+ ^g^ BIC_90_ 780 µg/mL	+ ^g^ BEC_90_ 1560 µg/mL		

Lipid targets: Mannosyldiinositolphosphorylceramide (M(IP)2C); glucosylceramide (GlcCer); phosphatidic acid (PA); phosphatidylinositol 3-phosphate PI(3)P; phosphatidylinositol 4-phosphate PI(4)P; phosphatidylinositol 5-phosphate PI(5)P; phosphatidylinositol 4,5-bisphosphate (PI(4,5)P2); phosphatidylinositol 3, 4, 5-triphosphate (PI(3,4,5)P3); phosphatidylserine (PS). Cells used: umbilical vein endothelial cells (HUVEC); human hepatoma (HepG2); adult human dermal fibroblast cells (AHDF); human peripheral blood mononuclear cells (PBMCs); human bronchial epithelial cells (Beas-2B); human embryonic kidney cells (HEK-293); rat lymphocyte (R8); human hepatocyte (HSP); Chinese hamster ovary (CHO) cells; breast epithelial cells (MCF 10A). BIC_50_—peptide concentration causing 50% inhibition of biofilm formation; BEC_50_—peptide concentration required to diminish pre-grown biofilms by 50%. Biofilm formation conditions used: ^a^ yeast cells (10^6^ cells/mL) were incubated at 37 °C for 24 h in MHB with 0.5% DMSO; ^b^ yeast cells (10^6^ cells/mL) were allowed to adhere for 60 min at 37 °C, after which the plates were gently washed and incubated at 37 °C for 24 h in MHB; ^c^ yeast cell culture (OD_600_ 0.1) was allowed to adhere for 60 min at 37 °C, after which the plates were gently washed and incubated at 37 °C for 24 h in RPMI-1640 with 0.5% DMSO; ^d^ yeast cells were incubated at 37 °C for 24 h in RPMI-1640; ^e^ yeast cells (10^6^ cells/mL) were incubated at 37 °C for 24 h in RPMI-1640; ^f^ yeast cells (10^8^ CFU/mL) were prepared in TSB and incubated for 48 h; ^g^ inoculum (0.5 McFarland) was incubated at 37 °C for 90 min, after which the plates were gently washed and incubated at 37 °C for 24 h.

**Table 3:** Immunomodulatory effects and related properties of plant defensins.

Defensin	Cell Lines/ Organisms	Concentration	Effects	Method Used	References
EgK5 (synthetic peptide)	Lewis rat ovalbumin-specific CD4^+^ T_EM_ cells transduced with GFP	0.1–100 nM	Suppression of antigen-driven T_EM_ cell proliferation	Incorporation of [^3^H] thymidine with consequent β-scintillation counting	[106]
	Collagen-induced arthritis and ovalbumin-induced dermatitis in Lewis rats	0.1 mg/kg	Reduction in clinical scores in collagen-induced arthritis, reduced ear thickness, and immune infiltration in ovalbumin-induced dermatitis	X-ray, hematoxylineosin staining	
γ-Thionin (*Capsicum chinense*)	bMECs	0.005–5 μg/mL	Up-regulation of TLR2 mRNA expression and membrane abundance of TLR2; increase in NO production; increased expression of genes coding TNFα, IL-1β, and IL-10	Real-time PCR, flow cytometry analysis, ELISA assay, Griess test	[107,108]
		0.1 μg/mL	Reduction in p38, ERK1/2, and Akt phosphorylation state; increase in JNK activity; activation of E2F-1, EGR, CBF, AP-1, MEF, and NF-1 and other transcription factors; decreased activity of histone demethylases		
	bMECs upon the *Staphylococcus aureus* infection	1 μg/mL	Reduction in *S. aureus* internalization into bMECs by 50%	Gentamicin protection assays, real-time PCR, flow cytometry analysis, ELISA assay, Griess test, tranSignal Protein/DNA array I	
		0.005–5 μg/mL	Up-regulation of TLR2 membrane abundance; decrease in NO production; increased expression of genes coding TNFα, IL-1β, IL-6, and IL-8/CXCL8; increase in DEFB1 secretion		
		0.1 μg/mL	Increase in p38 phosphorylation state and decrease in ERK1/2 and JNK activity; activation of protein kinase Akt; increased activity of histone demethylases		
		0.1 μg/mL	Activation of transcriptional factors of inflammatory response, highlighting EGR, E2F-1, AP-1, and MEF, which were turned off by *S. aureus*		
Lc-def	Monocytes	4 µM	Increase in the production of both pro- and anti-inflammatory cytokines (IFNα2, IL-12, IL-6, IL-1RA, IL-10, and IL-13), chemokines (MIG, MIP-1, and MCP-3), and growth factors (G-CSF, EGF, and PDGF)	Real-time PCR, the multiplex xMAP assay	[49]
	Macrophages		Increase in the production of inflammatory chemokines MIG, MCP-1, and MIP-1		
	CD4^+^ FoxP3^+^ T cells (Tregs)		Induction of the production of pro-inflammatory cytokines, chemokines, and growth factors (IL-17F, GM-CSF, IFN-γ, TNF-α, MCP-1, MIP-1, and IP-10), IL-6, and only one anti-inflammatory, IL-10		
	moDCs, CD4^+^ T-helper, and CD8^+^ T cytotoxic cells		The absence of significant changes in cytokine profiles		
	Caco-2-polarized monolayer		Insignificant changes in cytokine profiles; induction of expression of genes coding IL-1, IL-8/CXCL8, and HBD2		
Psd1	Murine lung metastatic melanoma model	1 mg/kg	Psd1 does not only inhibit the formation of lung metastasis nodules in a murine model in vivo, but also decreases the number of inflammatory cells in lung tissues	Histological analysis	[80]
	Caco-2-polarized monolayer	2 µM	Psd1 is able to pass through the Caco-2 cell monolayer	Trans-epithelial transport assay, using FITC-labelled Psd1	[79]
			Insignificant changes in cytokine profiles; increase in the production of IL-6, IL-8/CXCL8, and IP-10; induction of expression of genes coding IL-1β, IL-8/CXCL8, and HBD2	Real-time PCR, the multiplex xMAP assay	
	Caco-2-polarized monolayer upon the *Candida albicans* infection		Insignificant changes in cytokine profiles; induction of expression of genes coding IL-1β, IL-8/CXCL8, and HBD2; increase in the production of IL-6		
	Monocytes and moDCs (almost the same action)		Increases the production of pro-inflammatory (TNFα, IL-8/CXCL8, IL-12, IL-15, IP-10, MCP-1, MCP-3, MIG, MIP-1α, MIP-1β, and GM-CSF) anti-inflammatory (IL-5, IL-10, G-CSF, and IL-1RA) cytokines and chemokines, as well as cytokines with ambiguous action (IL-6 and IL-27)		
	Caco-2/moDCs co-culture		Insignificant induction of an immune response; a slight decrease in the production of IL-1β, IL-6, and MIG/CXCL9		
	Caco-2/monocytes co-culture		Insignificant induction of an immune response; increase in the production of IL-2, IL-10, MDC, and IL-17		
	Caco-2/moDCs co-culture upon the *Candida albicans* infection		Switching the immune response and mainly negating the stimulatory effects of pathogenic yeasts; inhibiting the production of TNFα, IL-6, IL-8/CXCL8, IL-12, IL-27, G-CSF, GM-CSF, and MIPs, induced by *C. albicans*		
	Caco-2/monocytes co-culture upon the *Candida albicans* infection		Switching the immune response and mainly negating the inhibitory effects of pathogenic yeasts; inducing the production of IL-8/CXCL8, IL-12, TNFα, G-CSF, and GM-CSF, inhibited by *C. albicans*		
PaDef (*Persea americana*) and γ-thionin	BUVEC and EA.hy926	5 ng/mL	Interferes with the vascular endothelial growth factor (VEGF) pathway, inhibiting processes related to angiogenesis in endothelial cells, such as proliferation, migration, and tube formation	Trypan blue dye, MTT assay, wound healing assay, matrigel induction	[109]
Ppdef1	Human nail model	10 mg/mL	Ppdef1 penetrates more effectively than such drugs as terbinafine and eficanazole	nail penetration assay, RP-HPLC	[66]
SolyC (synthetic peptide)	THP-1	50 μg/mL	No visible effects	ELISA assay, Griess test	[110]
	THP-1 stimulated with lipopolysaccharide		Promote anti-inflammatory effect by decreasing TNF-α and IFN-γ levels, as well as the production of NO_2_^−^		

Cells used: Bovine mammary epithelial cells (bMECs); human colorectal adenocarcinoma cells (Caco-2); CD4-positive FoxP3-positive T cells (CD4+ FoxP3+ T cells (Tregs)); CD4-positive T-helper cell (CD4+ T-helper cell); CD8-positive T cytotoxic cell (CD8+ T cytotoxic cell); monocyte-derived dendritic cells (moDCs); monocyte cell line from peripheral blood from an acute monocytic leukemia patient (THP-1); bovine endothelial cells (BUVEC); the human endothelial cell line (EA.hy926). DEFB1—bovine β-defensin 1; HBD2—human β-defensin 2.
